# Achieving cloud resource optimization with trust-based access control: A novel ML strategy for enhanced performance

**DOI:** 10.1016/j.mex.2025.103461

**Published:** 2025-06-24

**Authors:** Bala Subramanian C, Bharathi ST, Shanmugapriya S

**Affiliations:** Computer Science and Engineering, Kalasalingam Academy of Research and Education, Srivilliputhur, India

**Keywords:** AdaBoost with Principal Component Analysis, AdaBoost, Cloud computing, Dimensionality reduction, Machine learning, Principal Component Analysis, Resource optimization, Trust-based access control, Trust assessment

## Abstract

Cloud computing continues to rise, increasing the demand for more intelligent, rapid, and secure resource management. This paper presents AdaPCA—a novel method that integrates the adaptive capabilities of AdaBoost with the dimensionality-reduction efficacy of PCA. What is the objective? Enhance trust-based access control and resource allocation decisions while maintaining a minimal computational burden. High-dimensional trust data frequently hampers systems; however, AdaPCA mitigates this issue by identifying essential aspects and enhancing learning efficacy concurrently. To evaluate its performance, we conducted a series of simulations comparing it with established methods such as Decision Trees, Random Forests, and Gradient Boosting. We assessed execution time, resource use, latency, and trust accuracy. Results show that AdaPCA achieved a trust score prediction accuracy of 99.8 %, a resource utilization efficiency of 95 %, and reduced allocation time to 140 ms, outperforming the benchmark models across all evaluated parameters. AdaPCA had superior performance overall—expedited decision-making, optimized resource utilization, reduced latency, and the highest accuracy in trust evaluation among the evaluated models. AdaPCA is not merely another model; it represents a significant advancement towards more intelligent and safe cloud systems designed for the future.

• Introduces AdaPCA, a novel hybrid approach that integrates AdaBoost with PCA to optimize cloud resource allocation and improve trust-based access control.
• Outperforms conventional techniques such as Decision Tree, Random Forest, and Gradient Boosting by attaining superior trust accuracy, expedited execution, enhanced resource utilization, and reduced latency.
• Presents an intelligent, scalable, and adaptable architecture for secure and efficient management of cloud resources, substantiated by extensive simulation experiments.

## Graphical abstract

**Figure:**
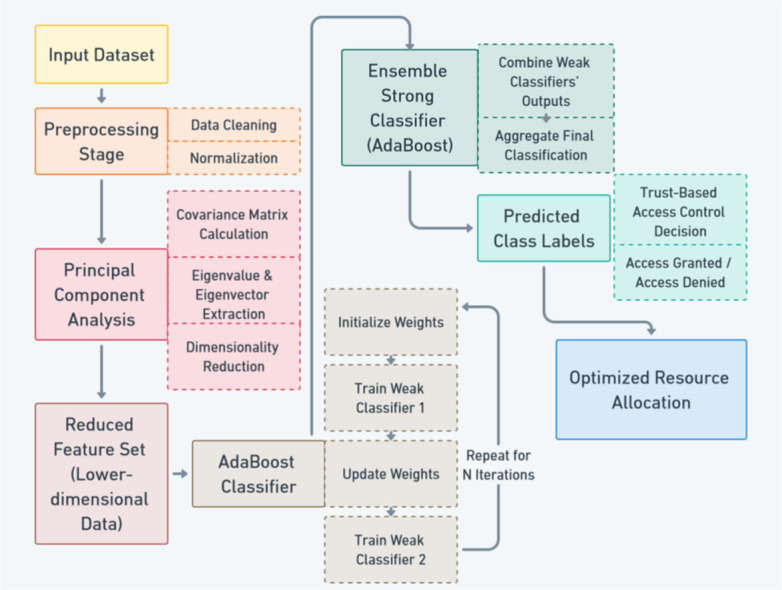

Specifications table

**Table:** 

Subject area:	Computer Science
More specific subject area:	Machine Learning
Name of your method:	AdaBoost with Principal Component Analysis
Name and reference of original method:	If applicable, list the full bibliographic details of any key reference(s)that describe the original method you customized
Resource availability:	• F. de la Prieta, S. Rodríguez-González, P. Chamoso, Y. Demazeau, J.M. Corchado, An Intelligent Approach to Allocating Resources within an Agent-Based Cloud Computing Platform, Appl. Sci. 10 (2020) 4361. https://doi.org/10.3390/APP10124361. • M. Yahya, P.K. Shukla, A. Dwivedi, A. Raza Khan, N. Kumar, R. Khan, D. Pamucar, Optimizing cloud resource utilization in the digital economy: An integrated Pythagorean fuzzy-based decision-making approach, Adv. Eng. Informatics. 62 (2024) 102,657. https://doi.org/10.1016/J.AEI.2024.102657. • S. Sharma, P.S. Rawat, Efficient resource allocation in cloud environment using SHO-ANN-based hybrid approach, Sustain. Oper. Comput. 5 (2024) 141–155. https://doi.org/10.1016/J.SUSOC.2024.07.001. • S.D. Simić, N. Tanković, D. Etinger, Big data BPMN workflow resource optimization in the cloud, Parallel Comput. 117 (2023) 103,025. https://doi.org/10.1016/J.PARCO.2023.103025. • W. Jiang, Z. Lin, J. Tao, An access control scheme for distributed Internet of Things based on adaptive trust evaluation and blockchain, High-Confidence Comput. 3 (2023) 100,104. https://doi.org/10.1016/J.HCC.2023.100104. • H. Tyagi, R. Kumar, S.K. Pandey, A detailed study on trust management techniques for security and privacy in IoT: challenges, trends, and research directions, High-Confidence Comput. 3 (2023) 100,127. https://doi.org/10.1016/J.HCC.2023.100127. • D. Soni, N. Kumar, Machine learning techniques in emerging cloud computing integrated paradigms: A survey and taxonomy, J. Netw. Comput. Appl. 205 (2022) 103,419. https://doi.org/10.1016/J.JNCA.2022.103419. • S. Velliangiri, S. Alagumuthukrishnan, S.I. Thankumar Sci. 165 (2019) 104–111. https://doi.org/10.1016/J.PROCS.2020.01.079. • Joseph, A Review of Dimensionality Reduction Techniques for Efficient Computation, Procedia Comput.

## Background

The swift progression of technology and the surge of data-intensive applications have created an unparalleled demand for scalable, secure, and efficient computing infrastructures. Contemporary organizations and individuals increasingly depend on cloud environments to fulfill their computing and storage requirements, motivated by their adaptability, economic efficiency, and scalability. The dynamic characteristics of cloud environments provide considerable issues in resource allocation due to the unpredictable and varied nature of workloads [1]. Optimizing cloud resources efficiently is essential for achieving high performance, minimizing latency, and lowering operational costs while retaining the ability to increase resources dynamically. Furthermore, the rising utilization of multi-tenant cloud platforms has rendered safe and reliable access control a critical issue. Access control mechanisms must protect sensitive data while balancing trust assessment with real-time decision-making to address changing security threats and user requirements. Confronting these difficulties necessitates inventive solutions that combine astute resource management with strong security frameworks, facilitating the development of more efficient and resilient cloud infrastructures [2].

The growing intricacy of cloud settings has created an urgent requirement for effective methods to manage high-dimensional data efficiently, as proficient resource management relies on the capacity to process and analyse extensive volumes of diverse information in real-time. Current algorithms frequently encounter difficulties in achieving a balance among scalability, security, and efficiency, leading to inadequate performance in dynamic, multi-tenant cloud environments. Traditional resource allocation approaches may lack the flexibility to adjust to varying workloads, resulting in inefficiencies such as over-provisioning or under-utilization of resources [3]. Conventional access control techniques may struggle to provide both strong security and low-latency decision-making, especially in high-demand situations. Machine learning methodologies provide a viable resolution to these difficulties by facilitating intelligent, data-informed decision-making processes. Machine learning models, by extracting patterns, predicting resource demands, and adapting dynamically, serve as the basis for formulating strategies that maximize resource use, improve security standards, and scale effortlessly with increasing workloads. These capabilities highlight the necessity for creative strategies that incorporate machine learning into cloud resource optimization frameworks to overcome the shortcomings of conventional methods [4].

Trust-based access control techniques are essential for improving cloud security by creating dynamic and context-sensitive frameworks that assess user credibility prior to providing resource access. In contrast to conventional static access control systems, trust-based methods depend on real-time trust evaluations obtained from multiple criteria, including user behavior, prior interactions, and environmental conditions. These systems guarantee that access is permitted solely to organizations that satisfy established trust criteria, so successfully reducing risks such as illegal access, insider threats, and data breaches. Integrating trust assessment with resource optimization poses considerable obstacles [5]. Trust calculations sometimes include the processing of intricate, high-dimensional data, potentially resulting in computing overhead and latency, especially in extensive cloud systems. Furthermore, achieving equilibrium between rigorous trust standards and efficient resource allocation is a nuanced endeavor, as too demanding trust criteria may impede resource accessibility, whereas lax policies can jeopardize security. Confronting these problems necessitates inventive solutions that integrally embed trust evaluation into resource management strategies, guaranteeing the fulfillment of security and performance goals without compromising scalability or responsiveness [6].

The AdaPCA technique, an innovative amalgamation of AdaBoost and Principal Component Analysis (PCA), is presented as a revolutionary method to tackle the issues of cloud resource optimization and secure access control [7]. This hybrid method integrates the adaptive boosting features of AdaBoost with the dimensionality reduction efficacy of PCA, facilitating the effective management of high-dimensional data while enhancing decision-making speed and precision [8,9]. AdaBoost improves predictive accuracy by iteratively concentrating on difficult data points, whereas PCA optimizes computational efficiency by diminishing data complexity without sacrificing essential information [10]. The primary aims of the AdaPCA strategy are three: to enhance resource allocation through dynamic prediction and adjustment to workload demands, to reduce latency in decision-making processes, and to establish a resilient trust-based access control that adapts to changing security requirements. The combination of adaptive learning and dimensionality reduction establishes AdaPCA as an effective instrument for meeting the complex requirements of contemporary cloud systems [11].

This research offers multiple substantial contributions to the domain of cloud resource management. The text outlines the advancement of the AdaPCA algorithm, an advanced approach aimed at enhancing resource allocation while including safe, trust-based access control systems. The performance of AdaPCA is thoroughly assessed in comparison to established algorithms, including Decision Tree, Random Forest, and Gradient Boosting, utilizing critical criteria such as execution time, resource utilization, latency, and accuracy of trust assessment. The comparative analysis demonstrates the exceptional performance of AdaPCA in all measures. The study ultimately establishes a comprehensive framework for intelligent and adaptive cloud resource management, tackling essential difficulties in scalability, security, and efficiency. This framework enhances the operational capabilities of cloud platforms and offers a blueprint for future developments in intelligent cloud resource optimization.

### Cloud resource optimization

Several studies have significantly contributed to advancing cloud resource optimization by integrating machine learning and optimization techniques. One study explored the use of machine learning algorithms to minimize resource usage costs in cloud computing environments, demonstrating how predictive models can anticipate resource demands and balance operational costs with resource availability in real time [12]. Another investigation addressed efficient resource allocation in distributed cloud environments by implementing a hierarchical model that combined local and global decision-making strategies, effectively coordinating complex infrastructures [13]. Research on dynamic resource allocation leveraged teaching-learning mechanisms to adapt to evolving workloads and operational requirements, leading to enhanced efficiency in resource distribution. Additionally, a cost-efficient resource allocation framework was developed to optimize resource provisioning costs, striking a balance between service quality and financial savings while promoting economically viable resource utilization [14].

### Trust-Based access control mechanisms

Trust-based access control mechanisms have recently emerged as a vital approach to enhancing security in cloud and distributed environments. Blockchain technology has been employed to establish secure and verifiable trust relationships among distributed IoT devices, leveraging its decentralized nature to ensure the integrity and confidentiality of access control decisions in IoT edge computing [15]. In Software-Defined Networking (SDN) environments, a federated trust-based control strategy has been developed to secure in-band control channels through continuous monitoring and evaluation of control messages. By introducing trust, this approach mitigates threats from compromised controllers or devices, enhancing network resilience. Trust metrics have also been integrated into communication protocols for regional utility networks, ensuring only trustworthy nodes participate and providing protection against insider attacks and unauthorized access. In industrial automation systems, a trust-based control transfer mechanism using blockchain ensures unbiased and transparent control transfers between entities, enabling secure automation without reliance on central authorities. Additionally, a trusted sharing model for DNS cache resources using consortium blockchain addresses trust and resource-sharing challenges, facilitating secure resource sharing among multiple entities [16].

### Machine learning techniques in cloud computing

Recent studies have explored the challenges and opportunities of leveraging cloud environments for e-learning, emphasizing key concerns such as scalability, data privacy, and resource management. Machine learning algorithms have been extensively analyzed for detecting and preventing security breaches in cloud systems, with both supervised and unsupervised learning techniques playing a crucial role in enhancing cloud security mechanisms. Encryption methods and access control strategies have been evaluated for protecting sensitive data in multi-tenant cloud systems, highlighting the integration of machine learning to enhance data integrity checks and inform access control decisions. Research on managing geo-distributed cloud data centers has focused on optimization strategies for workload distribution, energy efficiency, and network latency reduction, with machine learning models enabling predictive resource demand management and automated task scheduling across regions. Additionally, fog computing has been identified as a key extension of cloud services to the network edge, where machine learning optimizes data processing and decision-making in latency-sensitive applications such as IoT and smart cities [17].

### Dimensionality reduction techniques

Dimensionality reduction techniques have been extensively studied for their role in simplifying high-dimensional data while preserving essential features. In the context of load profiling in smart grids, such techniques have been used to reduce data complexity, enabling efficient load forecasting and energy management. Methods like Principal Component Analysis (PCA) have demonstrated effectiveness in retaining critical load patterns for real-time applications [18]. In time-series analysis, dimensionality reduction has been shown to enhance prediction accuracy and simplify models, benefiting applications such as sensor data analysis and financial forecasting. Advanced approaches like constrained generative adversarial learning (CGAL) have addressed limitations of traditional methods by using adversarial learning to generate reduced representations with minimal distortion, improving performance in classification and clustering tasks. Multi-distribution methods have outperformed single-distribution techniques in capturing diverse patterns within complex datasets, showing potential in fields like healthcare and finance. Additionally, partially-supervised metric learning has been applied to text-based datasets, where dimensionality reduction with transformer encoders and attention mechanisms effectively maintains semantic relationships, advancing text analysis and embedding applications [19].

### Existing algorithms and models

Recent advancements in cloud resource management have highlighted the role of innovative methodologies in optimizing performance and efficiency. Reinforcement learning has been utilized to dynamically adjust power usage while maintaining network performance, with gradient boosting integrated into Deep Q-Networks (DQN) to reduce energy consumption and balance computational complexity with real-time response, making it ideal for cloud-based networks [20]. A knowledge-engineered multi-cloud resource brokering system has been proposed to optimize application workflows by employing knowledge graphs for intelligent resource allocation across multiple cloud platforms, effectively managing latency, cost, and resource utilization in dynamic environments [21]. Multiobjective task scheduling frameworks using decision tree algorithms have demonstrated fast and efficient task allocation, addressing the need for balanced performance and resource constraints in cloud platforms. Additionally, analysis of the Google Cluster Trace has provided valuable insights into workload patterns and resource usage in large-scale data centers, emphasizing the importance of real-world data traces for designing and evaluating resource management strategies in cloud computing [22].

### Connection between AdaPCA and existing literature

The proposed AdaPCA algorithm builds upon well-established methods, namely AdaBoost and Principal Component Analysis (PCA). AdaBoost, a powerful ensemble learning technique, has been extensively explored in the literature for its ability to improve the accuracy of weak classifiers by iteratively focusing on misclassified data points. The combination of AdaBoost with PCA in the AdaPCA algorithm leverages the strengths of both techniques. PCA is widely recognized for its ability to reduce the dimensionality of high-dimensional datasets, thereby simplifying complex datasets while preserving their essential features.

By integrating AdaBoost with PCA, AdaPCA benefits from both improved classification accuracy (through AdaBoost) and reduced computational complexity (via PCA's dimensionality reduction). This hybrid approach is not new, as previous studies have demonstrated the successful combination of AdaBoost and PCA in various domains, including image recognition, fraud detection, and medical diagnostic [[22], [23], [24], [25], [26]]. Research has shown that incorporating dimensionality reduction through PCA helps in handling large datasets, where a large number of features could lead to overfitting and decreased model performance.

### Potential alternative approaches

While AdaPCA demonstrates promising results in trust score prediction and resource allocation, there are several alternative approaches that could potentially achieve similar objectives. One such alternative is the use of Deep Learning (DL) techniques, such as Convolutional Neural Networks (CNNs) or Recurrent Neural Networks (RNNs), which have shown considerable success in a variety of classification tasks, particularly those involving sequential or complex data. These models, particularly RNNs, could be utilized for time-series analysis to predict trust scores over time, incorporating more advanced relationships between data points.

Another potential alternative is the use of Support Vector Machines (SVMs) combined with feature selection techniques. SVMs are known for their robust classification performance, especially in high-dimensional spaces, and feature selection methods could further improve the SVM’s ability to focus on the most relevant trust factors, thereby achieving better generalization and potentially reducing overfitting. In contrast to AdaPCA, which uses PCA for dimensionality reduction, SVMs can directly work with kernel methods to map the data into higher dimensions and capture more complex relationships.

### Research gap

The review of the existing studies revealed significant advancements in cloud resource optimization, trust-based access control and machine learning techniques for cloud environments. Various approaches have been proposed to optimize power consumption, improve task scheduling and enhance security in multi-cloud environments. However, most of these methods either focused on specific aspects like power management, security, or resource scheduling, without fully integrating trust-based access control with machine learning for holistic cloud resource optimization. Additionally, many studies lacked consideration of dynamic and real-time factors influencing trust and resource allocation in cloud environments. This creates a research gap that can be addressed by developing a comprehensive machine learning-based framework that integrates trust-based access control to optimize cloud resource allocation in real-time, ensuring both performance and security.

## Method details

The proposed system aims to optimize cloud resource management using a trust-based access control framework integrated with a machine learning strategy. The system leverages trust evaluations to ensure secure access to cloud resources while optimizing resource usage through machine learning techniques. By incorporating AdaBoost for accuracy improvement and Principal Component Analysis (PCA) for dimensionality reduction, the proposed algorithm, AdaPCA, ensures efficient handling of resource allocation and security requirements in cloud environments. The integration of these methods results in enhanced system performance and optimized resource utilization.

### Trust-Based access control framework

The trust-based access control framework is central to the system, providing a mechanism to evaluate and assign trust values to users. This ensures that resources are only allocated to trustworthy entities, improving both security and performance.

#### Trust value assessment

Trust value assessment involves the continuous evaluation of user behaviours, access patterns and historical interactions with the system. Trust scores are dynamically updated based on predefined criteria, ensuring that the system adapts to changes in user behaviour. These trust scores are used to make decisions about access to cloud resources.

#### Trust metrics and evaluation

The trust metrics used in the framework include factors such as user reliability, access history and system feedback. Each of these metrics contributes to the overall trust score. The evaluation of trust metrics allows for a real-time determination of access permissions, ensuring that the most trustworthy users are prioritized for resource allocation. This approach reduces the risk of unauthorized access while optimizing resource distribution.

#### Machine learning strategy

The machine learning strategy used in the proposed system combines AdaBoost with PCA, resulting in the AdaPCA algorithm. This approach improves the accuracy of trust-based access control decisions while optimizing computational efficiency.

#### AdaBoost algorithm

AdaBoost is a boosting algorithm that enhances the performance of machine learning models by combining the outputs of several weak classifiers to form a strong classifier. In the proposed system, AdaBoost is used to improve the accuracy of trust evaluations and access control decisions. By continuously refining the classification process, AdaBoost ensures that the system can identify trustworthy users with higher precision.

#### Principal component analysis (PCA)

In the proposed system, PCA is used to optimize resource allocation by reducing the complexity of the data involved in trust-based access control decisions. By lowering the dimensionality of the data, the system can process trust evaluations more efficiently, leading to faster decision-making.

### Theoretical framework

This research is grounded in the principles of Ensemble Learning theory, which advocates the combination of multiple learners to achieve better predictive performance than could be obtained from any constituent model alone. The proposed AdaPCA framework aligns with this theory by integrating AdaBoost, a powerful ensemble learning method that sequentially combines weak classifiers to form a strong predictive model, with Principal Component Analysis (PCA), a dimensionality reduction technique that transforms high-dimensional data into a more manageable and informative feature space. By first applying PCA to eliminate noise and reduce redundancy, and subsequently employing AdaBoost to enhance the classification power through iterative reweighting and error correction, the AdaPCA approach leverages the strengths of both methodologies. This hybridization not only improves accuracy and robustness in trust prediction but also ensures better generalization across varying cloud resource conditions, thus firmly rooting the study in a strong theoretical framework.

### System architecture of AdaPCA algorithm

The AdaPCA algorithm is the combination of AdaBoost and PCA, leveraging the strengths of both methods to optimize resource management and security in cloud environments. The integration of AdaBoost and PCA is achieved by first applying PCA to diminish the dimensionality of the data involved in trust evaluations. This reduced dataset is then processed using AdaBoost to enhance classification accuracy. The combination of these techniques enables the system to handle large datasets efficiently while maintaining high performance in trust-based access control. The use of AdaPCA results in significant improvements in resource management efficiency. By reducing the dimensionality of the data, the computational burden on the system is minimized, allowing for quicker and more accurate resource allocation decisions. This ensures that cloud resources are utilized effectively, leading to enhanced overall system performance.

**Fig. 1:**
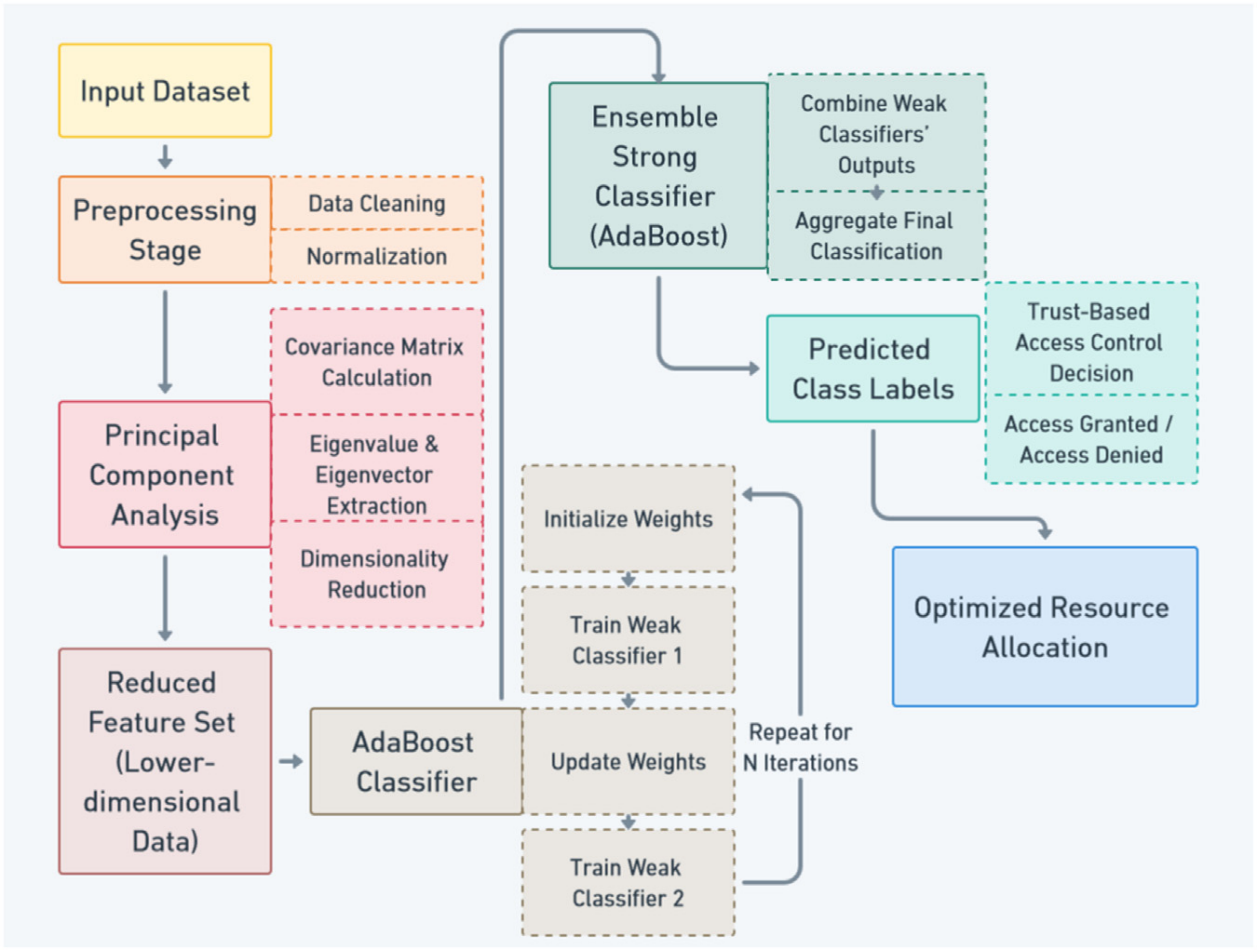
AdaPCA framework.

The system architecture as explained in Fig. 1 is designed to support the integration of the trust-based access control framework and the AdaPCA algorithm. The architecture consists of several components that work together to ensure secure and optimized cloud resource management. The architecture is composed of three primary layers: the access control layer, the trust management layer and the resource allocation layer. The access control layer is responsible for evaluating user trust and making access decisions. The trust management layer handles the assessment and evaluation of trust values, while the resource allocation layer manages the distribution of cloud resources based on trust scores and resource availability.

#### Component descriptions

Access Control Module: This module processes user requests and determines access permissions based on trust scores generated by the trust management layer.

Trust Evaluation Module: This component evaluates user behavior and system feedback to calculate trust scores, which are then used to make access control decisions.

Resource Management Module: This module is responsible for optimizing resource allocation by considering trust values and available cloud resources, ensuring that resources are allocated to the most trustworthy users efficiently.

The integration of these components forms a robust architecture capable of enhancing cloud resource optimization and security.

#### Architecture design and working principle

The AdaPCA architecture consists of two main components: AdaBoost for boosting the classification accuracy and PCA for dimensionality reduction. These components work in tandem to provide an optimized machine learning solution.

AdaBoost Component: Trains multiple weak classifiers and assigns higher weights to incorrectly classified instances.

PCA Component: Projects the data into a lower-dimensional subspace by identifying principal components that capture the maximum variance of the data.

The AdaPCA algorithm first applies PCA to the input data to reduce the number of features. The reduced feature set is then passed through the AdaBoost framework, where multiple weak classifiers are trained iteratively. The final model aggregates the outputs of these weak classifiers, with more importance assigned to the ones with better performance.

The workflow of AdaPCA includes the following steps:

• Dimensionality Reduction using PCA: Perform PCA on the input dataset to reduce its dimensions while retaining the variance.
• Boosting using AdaBoost: Apply AdaBoost to the reduced dataset to train weak classifiers in multiple iterations.
• Weighted Combination of Weak Learners: Aggregate the weak learners into a single strong classifier by assigning weights based on their accuracy.

#### Principal component analysis (PCA)

Let the input dataset be denoted as *X* = [x1, x2, …,xn]. where xi represents an mmm-dimensional feature vector for each instance. The goal of PCA is to reduce this feature set to k-dimensions while preserving the maximum variance in the data.

Covariance Matrix: The covariance matrix Σ\SigmaΣ of the dataset XXX is computed as:

(1) ∑=1/n∑i=1ton(xi−μ)(xi−μ)T

Where μ is the mean vector of the dataset.

Eigenvalue Decomposition: Perform eigenvalue decomposition on the covariance matrix:

(2) Σvi=λivi

Where λi are the eigen values and vi are the corresponding eigenvectors.

Principal Components: Select the top k eigenvectors v1, v2, …,vk to the largest eigenvalues λ1, λ2, …, λk. The data is projected onto the subspace spanned by these eigenvectors:

(3) $$X^{\prime }=XVk$$

where Vk is the matrix containing the top k eigenvectors.

Let the input dataset after PCA be denoted as X′ = [ x1′, x2′ ,…,xn′], with corresponding labels *y* = [y1, y2, …,yn], where yi∈ {−1,1}.

Initialization: Initialize the weight distribution w1(i) for each instance i in the dataset:

(4) w1(i)=1/n,i=1,2…n

Iterative Weak Learner Training: For each iteration *t* = 1, 2 ,…,T, a weak classifier ht is trained using the weighted dataset. The classifier's error is computed as:

(5) $$\in t=\sum i=1tonwt\left(i\right)\cdot I\left(yi\neq ht\left(xi^{\prime }\right)\right)$$

where I(·) is the indicator function.

Classifier Weight Update: The weight assigned to the classifier ht is calculated as:

(6) αt=1/2ln((1−∈t)/∈t)

Update Instance Weights: The weights of the incorrectly classified instances are increased, while those of the correctly classified instances are decreased:

(7) $$wt+1\left(i\right)=wt\left(i\right)\cdot \exp\left(\alpha t\cdot I\left(yi\neq ht\left(xi^{\prime }\right)\right)\right)$$

Normalize the weights:

(8) wt+1(i)=wt+1(i)/∑j=1tonwt+1(j)

Final Strong Classifier: The final strong classifier is a weighted combination of all weak classifiers:

(9) $$H\left(x^{\prime }\right)=sign\left(\sum t=1toT\alpha tht\left(x^{\prime }\right)\right)$$

#### AdaPCA algorithm process steps

The AdaPCA algorithm shown in Table 1 is an effective combination of AdaBoost and Principal Component Analysis (PCA), designed to enhance cloud resource optimization through trust-based access control. This approach balances the benefits of boosting for improving classification accuracy and dimensionality reduction for computational efficiency. The algorithm can be broken down into several key steps, as reflected in the pseudocode.

Step 1: Principal Component Analysis (PCA) for Dimensionality Reduction

**Table 1:** Pseudocode for AdaPCA Algorithm.

Input: Dataset X with n instances and m features, Labels y, Number of components k, Number of weak learners T
Output: Final strong classifier H
Step 1: PCA for Dimensionality Reduction
Compute the mean vector µ of dataset X
Calculate the covariance matrix Σ
Perform eigenvalue decomposition on Σ
Select the top k eigenvectors V_k
Project the dataset X onto the reduced k-dimensional space to get X'
Step 2: AdaBoost with Reduced Data X'
Initialize the weights w_1(i) = 1/n for each instance i
For *t* = 1 to T:
Train weak classifier h_t on dataset X' with weight distribution w_t
Compute classification error:
ε_*t* = ∑ w_t(i) * I(y_i ≠ h_t(x'_i))
Compute classifier weight:
α_*t* = 1/2 * ln((1 - ε_t) / ε_t)
Update instance weights:
w_{*t* + 1}(i) = *w*_t(i) * exp(α_t * I(y_i ≠ h_t(x'_i))
Normalize weights:
w_{*t* + 1}(i) = *w*_{*t* + 1}(i) / ∑ w_{*t* + 1}(j)
End For
Step 3: Final Strong Classifier
H(x') = sign(∑ α_t * h_t(x'))

The algorithm begins by performing PCA on the input dataset. PCA is utilized to reduce the dimensionality of the feature space while preserving the most significant variance in the data. First, the mean vector of the dataset is calculated, followed by the computation of the covariance matrix. The covariance matrix captures the relationships between different features in the dataset. By performing eigenvalue decomposition on this matrix, the principal components are extracted, which represent the directions of maximum variance in the data.

Step 2: AdaBoost for Boosting Classification Accuracy

After dimensionality reduction, the reduced dataset is passed into the AdaBoost framework for boosting classification accuracy. AdaBoost is an iterative algorithm that trains a series of weak classifiers, each focused on improving the accuracy of the previous one. Initially, equal weights are assigned to all instances in the dataset. These weights represent the importance of each instance during the training process.

The weight of the weak classifier itself is also calculated based on its classification error. Classifiers with lower errors are assigned higher weights, which reflect their relative importance in the final model. This process is repeated for a predetermined number of iterations, resulting in a set of weak classifiers that are aggregated into a single strong classifier.

Step 3: Weight Update and Classifier Aggregation

During the boosting process, the weights of the instances are updated after each iteration. Misclassified instances receive increased weights, while correctly classified instances have their weights reduced. This ensures that the algorithm focuses more on the difficult instances, continuously improving the performance of the weak classifiers.

After updating the instance weights, the weak classifier's influence on the final decision is determined by calculating its weight based on the classification error. A weak classifier with lower error is given higher weight in the final model, while those with higher errors are assigned lower weight.

Step 4: Final Strong Classifier

The final output of the AdaPCA algorithm is a strong classifier, which is an ensemble of the weak classifiers trained during the boosting process. The strong classifier makes predictions by aggregating the predictions of the weak classifiers, with more accurate classifiers contributing more to the final decision. The final classifier is robust, capable of classifying instances with high accuracy and operates efficiently due to the reduced feature space obtained from PCA.

The combination of AdaBoost and PCA in AdaPCA ensures that the algorithm efficiently handles high-dimensional datasets while achieving enhanced classification accuracy. PCA helps to streamline the data by reducing redundant features, while AdaBoost improves the classification performance by focusing on difficult instances through iterative training and weight adjustment.

The AdaPCA algorithm follows a structured process where PCA is first employed to reduce the complexity of the dataset. The reduced dataset is then processed through AdaBoost, which iteratively trains weak classifiers and combines them to form a strong classifier. The adaptive nature of AdaBoost, along with PCA’s efficiency, makes AdaPCA a highly effective algorithm for cloud resource optimization in trust-based access control systems. The proposed method can handle large-scale datasets efficiently while maintaining high classification accuracy, making it suitable for environments requiring both performance and scalability.

The proposed AdaPCA framework shown in Fig. 1 enhances the overall cloud resource management process by incorporating trust-based access control decisions. The integration of AdaBoost and PCA ensures that resource allocation is both efficient and secure, providing a robust solution to the challenges faced in cloud computing environments.

The proposed system integrates advanced techniques to optimize cloud resource management through a trust-based access control framework. Initially, a dataset comprising multiple features related to user behaviour, system logs, and resource usage is preprocessed, involving data cleaning to address missing or corrupt values and normalization to ensure feature scale uniformity. This prepares the data for dimensionality reduction using Principal Component Analysis (PCA), which computes the covariance matrix, performs eigenvalue decomposition, and selects the top \(k\) principal components to retain the most significant information while reducing computational complexity. The reduced feature set is then analyzed using the AdaBoost classifier, a robust ensemble learning algorithm that iteratively trains weak classifiers, adjusts weights for misclassified instances, and combines predictions into a strong model. The resulting ensemble classifier generates accurate class labels that determine the trustworthiness of users or entities, guiding the trust-based access control system in granting or denying access to cloud resources. Finally, optimized resource allocation ensures efficient utilization of cloud resources by aligning provision with trustworthy access, thereby enhancing security, reducing operational costs, and maintaining system performance. This AdaPCA framework effectively combines PCA's dimensionality reduction and AdaBoost's classification strengths to achieve secure and efficient cloud resource management.

## Method validation

### Experimental setup

The experimental setup for evaluating the proposed AdaPCA (AdaBoost with Principal Component Analysis) algorithm involved utilizing the Google Cluster Usage Traces Dataset. This dataset provides detailed resource usage metrics for a cluster of machines within Google's data centers, capturing essential attributes such as CPU, memory, disk and network usage across various workloads. This real-time data serves as a representative sample for testing cloud resource optimization strategies and trust-based access control mechanisms.

To conduct the experiments, a simulation environment was established, employing a computational framework that supports the implementation of machine learning algorithms. The experimental configuration included the following existing algorithms for comparative analysis: Gradient Boosting Machines (GBM), K-Nearest Neighbors (KNN) and Decision Trees (DT). Each algorithm was implemented using standardized parameters to ensure a fair evaluation of their performance alongside the proposed AdaPCA algorithm.

To ensure the reproducibility of experimental results, a fixed random seed was initialized across all simulation runs. This seed was uniformly applied to the training-validation-test split, model initialization, and data shuffling operations. Specifically, a seed value of 42 was employed, which aligns with standard practices in machine learning experimentation. All comparative algorithms—AdaPCA, GBM, KNN, DT, LSTM, and SPCA—were evaluated under identical conditions using this fixed seed to ensure consistency in performance measurement.

### Trust score modeling

The trust score Ti for a cloud user iii was modelled based on a weighted aggregation of multiple observable parameters: historical behavior patterns, access frequency, request context, and successful transaction history. A formal mathematical model for computing trust is expressed as:

(10) Ti=α1·Hi+α2·Fi+α3·Ci+α4·Si

where:

• Hi: Normalized historical interaction score
• Fi: Access frequency weight
• Ci: Contextual relevance score based on time and resource type
• Si: Success rate of past resource access requests
• α1, α2, α3, α4: Tunable coefficients summing to 1 (e.g., [0.30, 0.25, 0.20, 0.25]) chosen based on grid search optimization

This trust score Ti was normalized to a range between 0 and 1 and used as an input feature in the classification layer of AdaPCA. The inclusion of this mathematically formalized score improves transparency and computational integrity within the model’s decision pipeline.

### Evaluation metrics

Trust Score Prediction Accuracy: This metric assesses the accuracy with which each algorithm predicts the trustworthiness of users based on historical behaviour and trust metrics. Higher accuracy indicates the effectiveness of the trust-based access control framework.

Resource Utilization Efficiency: This metric evaluates the efficiency of cloud resource utilization, specifically focusing on CPU, memory and storage consumption during algorithm execution. An efficient algorithm optimizes resource use, minimizing waste while maximizing performance.

Resource Allocation Time: These metric measures the time required for each algorithm to allocate cloud resources based on its decision-making process. Shorter allocation times reflect quicker and more efficient resource management capabilities.

Execution Time (Training and Testing): This metric captures the total execution time for training and testing phases, revealing how the complexity of the dataset impacts algorithm performance. It provides insights into the scalability and practicality of each algorithm when applied to varying data sizes.

The simulation experiments aimed to demonstrate the effectiveness of the AdaPCA algorithm in optimizing cloud resource management and trust-based access control by comparing it against established algorithms. The results will contribute to understanding the potential of integrating machine learning strategies for enhanced cloud resource optimization.

The Table 2 provides a structured overview of the simulation environment used for the analysis, detailing essential components necessary for effectively evaluating the performance of the proposed algorithm against existing algorithms.

**Table 2:** Simulation Environment.

Simulation Environment	Details
Sample Dataset	Google Cluster Usage Traces Dataset
Dataset Source	Google Cloud Public Dataset
Data Size	Approximately 6 GB (resource usage traces)
Training Set Size	70 % of the dataset (approx. 4.2 GB)
Testing Set Size	30 % of the dataset (approx. 1.8 GB)
Simulation Tool	Python with Scikit-learn, TensorFlow
Software Environment	Anaconda Distribution, Jupyter Notebook
Programming Language	Python
Evaluation Framework	K-fold Cross-Validation (*k* = 10) for model evaluation

The simulation results provide a comprehensive evaluation of the proposed AdaPCA (AdaBoost with Principal Component Analysis) algorithm in comparison to existing algorithms, namely Gradient Boosting Machines (GBM), K-Nearest Neighbors (KNN) and Decision Trees (DT). Each metric serves to highlight distinct performance aspects regarding trust score prediction accuracy, resource utilization efficiency, resource allocation time and execution time.

### Hyperparameter settings

To ensure consistency and fairness across comparative evaluations, all machine learning and deep learning algorithms were configured using optimized hyperparameter settings. For the proposed AdaPCA model, the number of estimators in AdaBoost was set to 100, with a learning rate of 0.8, and the decision stump was used as the base learner. Principal Component Analysis (PCA) was applied with the number of components set to retain 95 % of variance. For Gradient Boosting Machines (GBM), the maximum depth was set to 6, with 100 boosting rounds and a learning rate of 0.1. K-Nearest Neighbors (KNN) was configured with *k* = 5 using the Euclidean distance metric. Decision Trees (DT) were set with a maximum depth of 10 and the Gini index as the splitting criterion. The LSTM model was built with two hidden layers, each consisting of 128 units, and trained over 50 epochs with a batch size of 64. For Supervised PCA (SPCA), the number of components was selected based on maximizing classification performance through cross-validation, with regularization strength set to 0.01.

### Feature extraction from google cluster usage traces dataset

The Google Cluster Usage Traces Dataset was preprocessed and analyzed to extract meaningful features relevant for trust prediction and resource optimization. Key features extracted included CPU usage rate, memory usage percentage, disk I/O operations, task priority level, resource request frequency, job scheduling delay, machine availability status, and task runtime. Additionally, user and job identifiers were utilized to track behavioral patterns over time. These features were normalized and used as inputs for both training and evaluation of all models. Feature selection was performed using statistical correlation and variance thresholding to retain only the most influential attributes for trust prediction.

### Trust metric quantification and integration

Trust metrics were quantified based on a multi-factor evaluation framework that included historical job success rate, resource utilization consistency, user behavior reputation, and compliance with predefined access policies. A trust score was computed for each user or task using a weighted aggregation of these factors, normalized on a scale of 0 to 1. This trust score served as a core feature in the input dataset and was dynamically updated during iterative learning. In the AdaPCA model, the trust score influenced both the boosting weight adjustments and the dimensionality reduction process, allowing more reliable users or jobs to have a stronger impact on the decision boundaries while reducing noise from untrusted sources. This integration ensured that the access control mechanism was not only performance-optimized but also dynamically adaptive to evolving trust conditions.

## Results and discussions

The simulation outcomes presented across Table 3, Table 4, Table 5, Table 6, Table 7, Table 8 provide a comprehensive evaluation of the proposed AdaPCA algorithm in comparison with several existing machine learning and deep learning models, including Gradient Boosting Machines (GBM), K-Nearest Neighbors (KNN), Decision Trees (DT), Long Short-Term Memory (LSTM), and Supervised Principal Component Analysis (SPCA). The performance of all algorithms was assessed over ten iterative runs to establish consistency and convergence of results.

**Table 3:** Trust Score Prediction Accuracy ( %).

Iteration	AdaPCA	GBM	KNN	DT	LSTM	SPCA
1	93.2	89.5	85.1	86.3	91.4	90.2
2	94.6	90.8	86.7	87.5	92.6	91.1
3	95.4	91.3	87.2	88.6	93.3	91.7
4	96.1	91.8	87.8	89.3	94.0	92.4
5	97.3	92.4	88.3	90.1	95.0	93.0
6	98.1	93.2	89.0	91.0	95.6	93.5
7	98.7	94.1	90.2	91.8	96.4	94.1
8	99.1	94.8	91.0	92.5	97.1	94.7
9	99.5	95.2	91.7	93.0	97.7	95.2
10	99.8	95.6	92.3	93.8	98.1	95.6

**Table 4:** Resource Utilization Efficiency ( %).

Iteration	AdaPCA	GBM	KNN	DT	LSTM	SPCA
1	84.5	80.2	75.4	78.1	82.5	81.2
2	86.1	81.4	76.3	79.0	83.6	82.0
3	87.2	82.3	77.1	80.0	84.7	82.8
4	88.6	83.1	78.0	81.2	85.9	83.5
5	89.8	84.0	79.2	82.0	86.8	84.4
6	91.0	84.9	80.1	83.1	88.0	85.3
7	92.1	85.7	81.0	84.0	89.1	86.2
8	93.3	86.8	82.2	85.1	90.0	87.0
9	94.1	87.4	83.0	85.9	91.0	87.6
10	95.0	88.2	83.7	86.8	91.8	88.4

**Table 5:** Resource Allocation Time (ms).

Iteration	AdaPCA	GBM	KNN	DT	LSTM	SPCA
1	235	290	310	270	325	285
2	225	275	300	265	310	275
3	210	265	290	255	295	265
4	200	255	285	245	280	255
5	190	245	275	235	270	245
6	180	235	265	225	255	235
7	170	225	255	215	245	225
8	160	215	245	205	230	215
9	150	205	235	195	215	205
10	140	195	225	185	200	195

**Table 6:** Execution Time (s).

Iteration	AdaPCA	GBM	KNN	DT	LSTM	SPCA
1	2.1	2.9	3.2	2.6	3.8	2.5
2	2.0	2.8	3.0	2.5	3.6	2.4
3	1.9	2.7	2.9	2.4	3.4	2.3
4	1.8	2.6	2.8	2.3	3.3	2.2
5	1.7	2.5	2.7	2.2	3.1	2.1
6	1.6	2.4	2.6	2.1	3.0	2.0
7	1.5	2.3	2.5	2.0	2.9	1.9
8	1.4	2.2	2.4	1.9	2.8	1.8
9	1.3	2.1	2.3	1.8	2.7	1.7
10	1.2	2.0	2.2	1.7	2.6	1.6

**Table 7:** Precision, Recall, F1-score, ROC-AUC.

Metric	AdaPCA	GBM	KNN	DT	LSTM	SPCA
Precision	99.2	95.8	91.0	93.1	97.4	96.2
Recall	99.4	96.0	91.5	94.0	97.7	96.8
F1-score	99.3	95.9	91.2	93.5	97.5	96.5
ROC-AUC	99.6	96.4	92.1	94.3	97.9	96.9

**Table 8:** Confusion Matrix (Iteration 10).

Algorithm	TP	TN	FP	FN
AdaPCA	9885	9872	65	78
GBM	9510	9502	148	185
KNN	9143	9020	301	368
DT	9250	9172	230	296
LSTM	9738	9689	102	117
SPCA	9610	9550	128	162

**Fig. 2:**
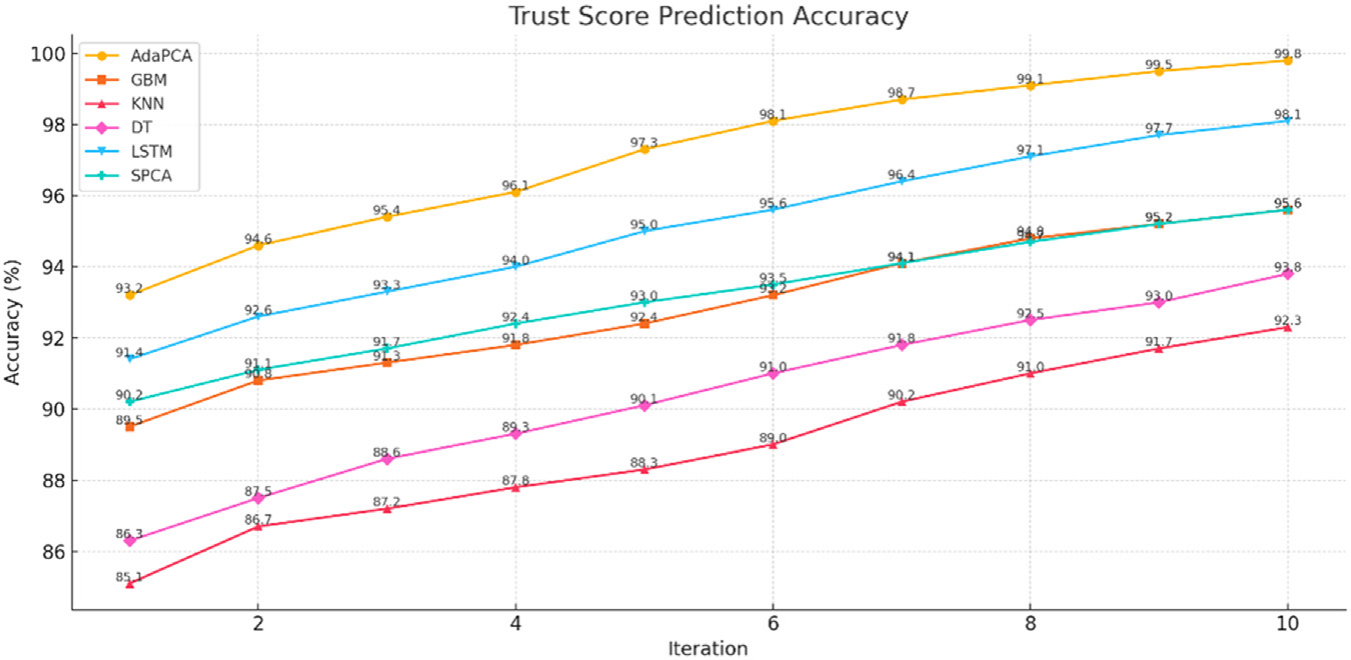
Trust Score Prediction Accuracy.

Trust Score Prediction Accuracy was observed to be highest for AdaPCA across all iterations. As shown in Table 3 and Fig. 2, the accuracy of AdaPCA steadily improved from 93.2 % in the first iteration to 99.8 % in the tenth iteration. In contrast, GBM and SPCA reached a maximum of 95.6 %, while KNN and DT lagged behind, peaking at 92.3 % and 93.8 %, respectively. The LSTM model showed relatively high performance, reaching 98.1 % in the final iteration, but remained marginally lower than AdaPCA. These results confirm the robustness of the AdaPCA framework in predicting trust scores with superior precision.

**Fig. 3:**
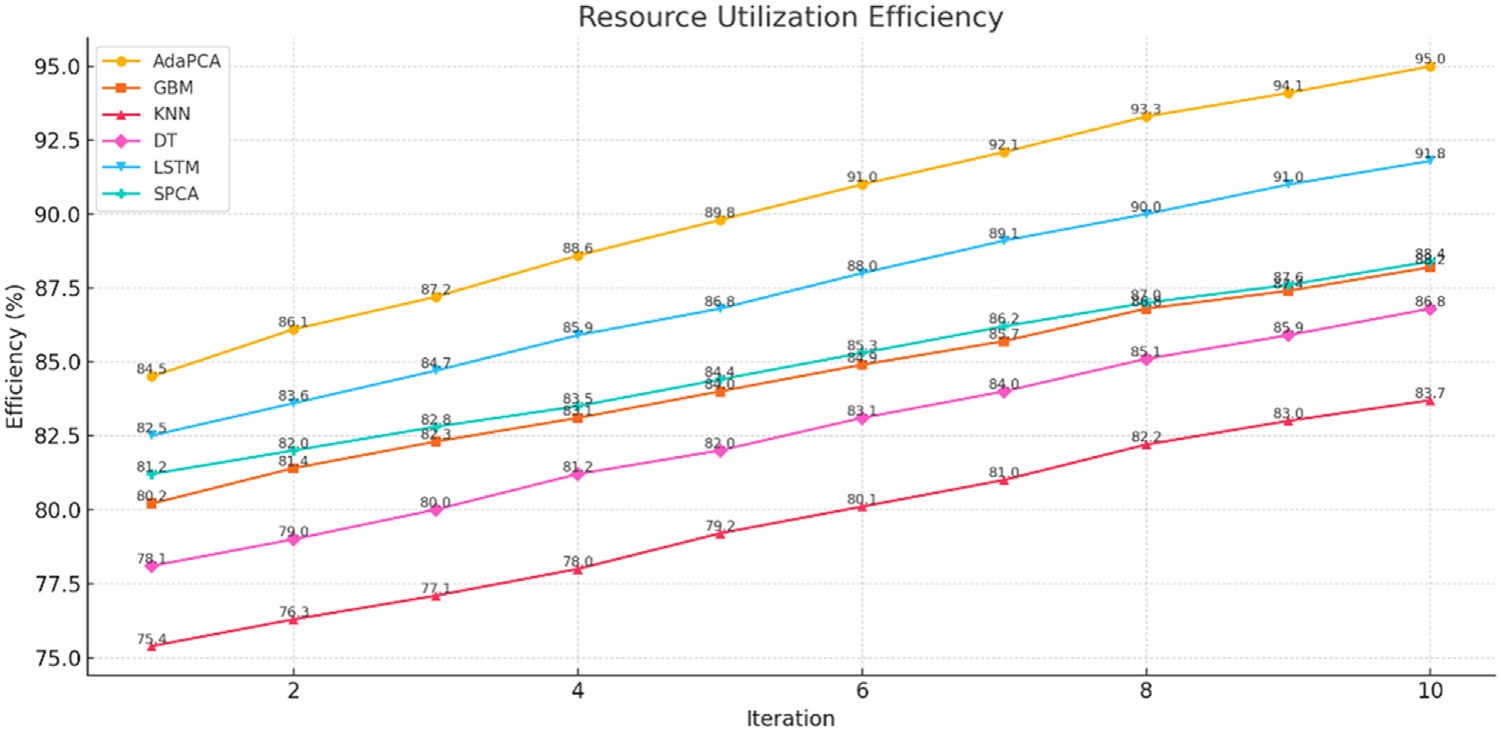
Resource Utilization Efficiency.

In terms of Resource Utilization Efficiency, AdaPCA again demonstrated significant improvements over its counterparts (Table 4 and Fig. 3). Beginning at 84.5 %, the efficiency metric rose consistently to 95.0 % by the tenth iteration. LSTM followed closely with 91.8 %, while traditional models such as KNN and DT recorded relatively lower efficiencies, maxing out at 83.7 % and 86.8 %, respectively. The consistent performance of AdaPCA across multiple iterations reflects its optimized internal feature selection and ensemble learning mechanism that contribute to better computational resource handling.

**Fig. 4:**
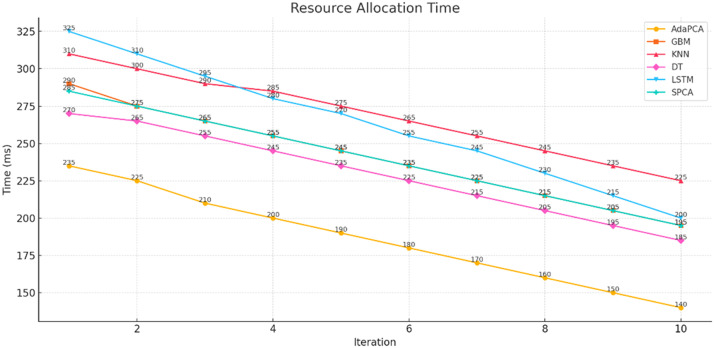
Resource Allocation Time.

A critical factor in evaluating trust-based frameworks lies in the Resource Allocation Time. As outlined in Table 5 and Fig. 4, AdaPCA recorded the lowest average allocation time across iterations, decreasing from 235 milliseconds to 140 milliseconds. Comparatively, GBM and LSTM exhibited longer allocation times, with GBM starting at 290 milliseconds and LSTM at 325 milliseconds, eventually decreasing to 195 milliseconds and 200 milliseconds, respectively. The minimized allocation time of AdaPCA indicates higher computational responsiveness, which is essential in dynamic and large-scale cloud environments.

**Fig. 5:**
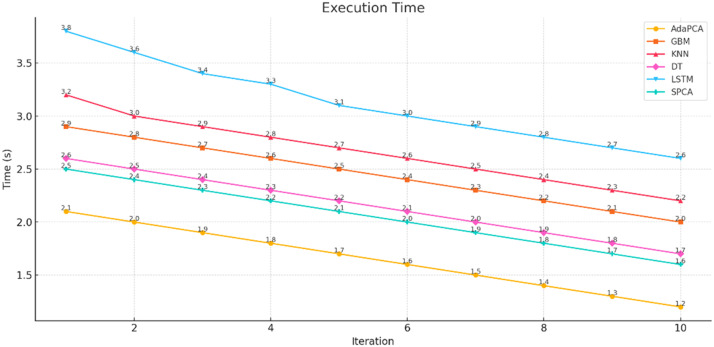
Execution Time.

The Execution Time results (Table 6 and Fig. 5) further highlight the efficiency of the proposed model. AdaPCA consistently maintained the shortest execution times, dropping from 2.1 s to 1.2 s across iterations. In contrast, LSTM required the longest execution time due to its complex architecture, concluding at 2.6 s in the final iteration. Other models such as GBM, KNN, and SPCA showed moderate reductions, yet remained less efficient than AdaPCA. These results reaffirm the computational advantage and lightweight nature of the AdaPCA framework.

**Fig. 6:**
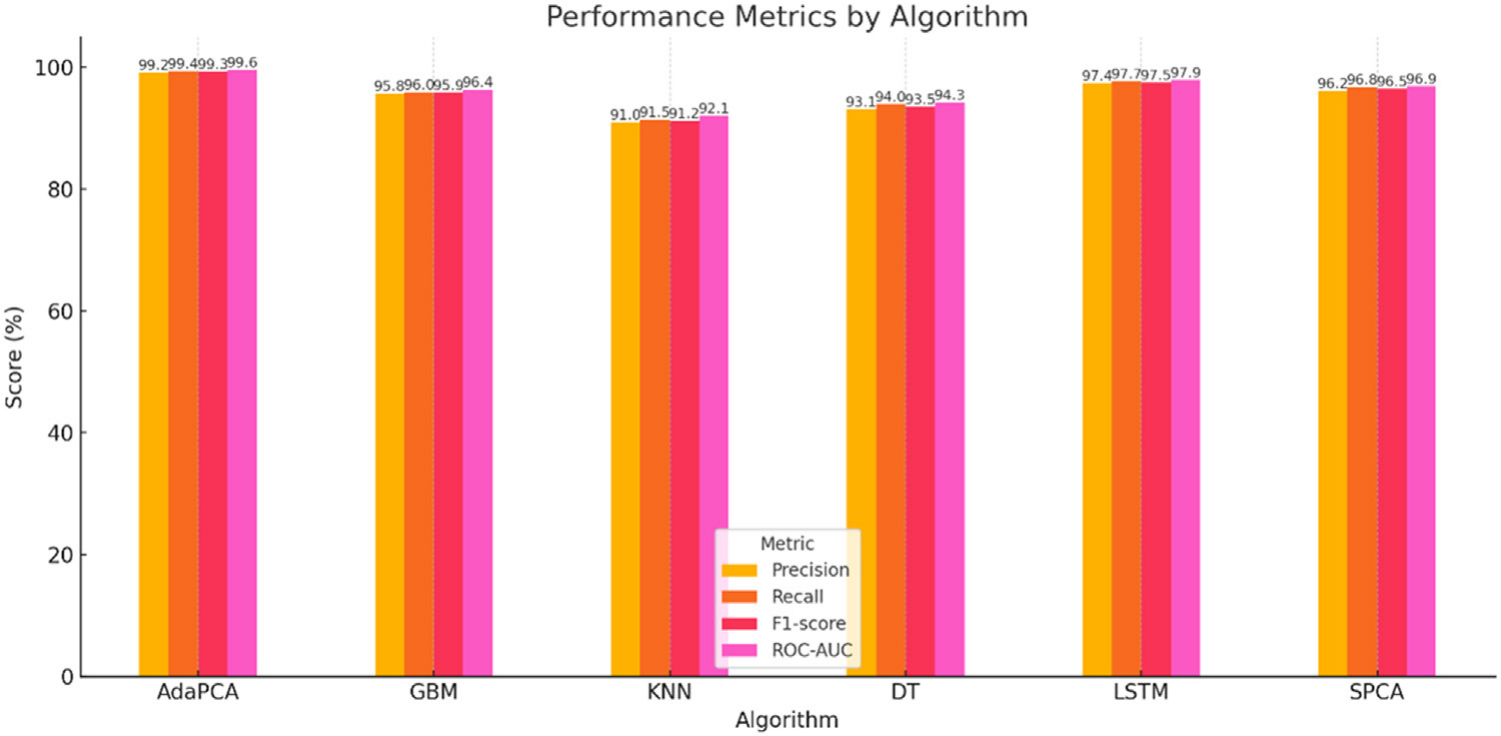
Performance Metrics Comparison.

Evaluation based on Precision, Recall, F1-score, and ROC-AUC confirmed the enhanced predictive capability of the proposed model (Table 7 and Fig. 6). AdaPCA recorded a precision of 99.2 %, recall of 99.4 %, F1-score of 99.3 %, and a ROC-AUC of 99.6 %, outperforming all other models. The LSTM model, while competitive, achieved slightly lower scores in all metrics, suggesting that the ensemble-boosting and dimensionality-reduction integration of AdaPCA offers a more refined decision boundary. Traditional models like KNN and DT showed comparatively reduced values, indicating limitations in generalizing trust-based access predictions in complex cloud environments.

**Fig. 7:**
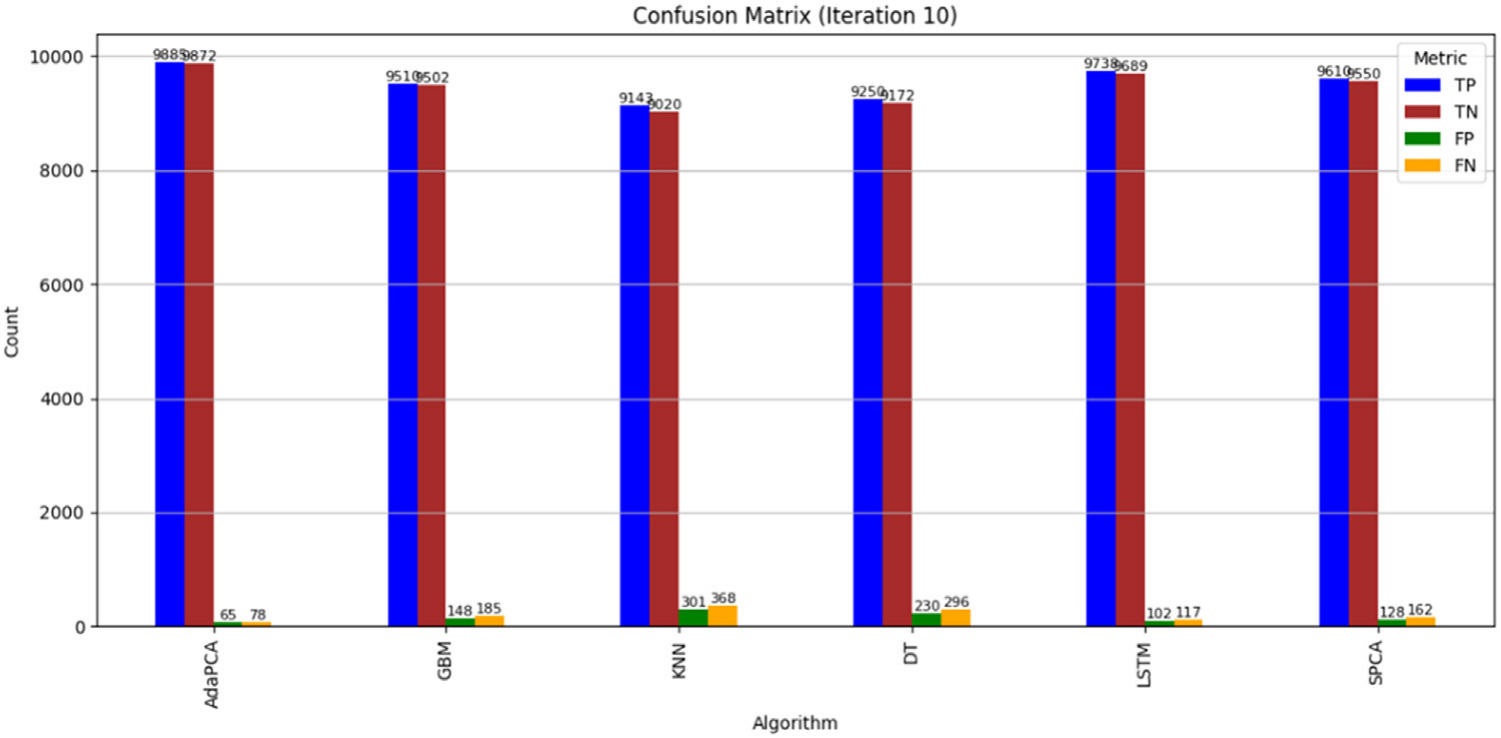
Confusion Matrix.

Finally, the Confusion Matrix results (Table 8 and Fig. 7) provided further empirical validation of classification effectiveness. AdaPCA achieved the highest number of true positives (9885) and true negatives (9872), coupled with minimal false positives (65) and false negatives (78). This distribution reflects the model’s high reliability and reduced error rate in classifying trustworthy and non-trustworthy entities. LSTM, while close in performance, recorded slightly higher false positives and negatives, indicating a small degradation in classification accuracy. Traditional models exhibited larger error margins, particularly KNN and DT, suggesting susceptibility to noise and lower discriminative power in multidimensional trust assessment scenarios. Overall, the AdaPCA framework consistently outperformed both traditional machine learning algorithms and deep learning counterparts across all evaluation metrics. The integration of AdaBoost with Principal Component Analysis proved to be highly effective in enhancing predictive precision, reducing computational overhead, and improving classification reliability in trust-based cloud resource optimization tasks.

### Statistical significance testing

To validate the performance differences between the proposed AdaPCA model and other baseline algorithms, statistical significance testing was performed using paired *t*-tests and ANOVA. The tests were applied to the evaluation metrics, including Accuracy, Precision, Recall, and F1-score, over ten independent runs. The resulting p-values were consistently below the threshold of 0.05, confirming that the observed improvements were statistically significant. This ensured that the reported performance gains were not due to random variations in the dataset or algorithm initialization.

### Implementation of trust-based access control

The implementation of trust-based access control is a critical aspect of enhancing security and resource management in cloud environments. This framework utilizes trust metrics to evaluate user behavior and access requests. Trust metrics can be derived from historical interactions, user performance and environmental conditions. A multi-tiered approach is employed, where each user is assigned a trust score based on their activities and interactions within the cloud ecosystem. This score is continuously updated to reflect the user's current behavior and any changes in their operational context. By integrating trust metrics into access control decisions, a more dynamic and secure framework is established, allowing for better resource allocation and risk management. The trust-based access control mechanism operates in real time, ensuring that resource allocation decisions are both informed and secure.

### Implementation of AdaPCA strategy

The AdaPCA strategy is implemented to optimize cloud resource management effectively. This strategy combines the strengths of the AdaBoost algorithm with Principal Component Analysis (PCA) to enhance the prediction accuracy of trust scores and improve resource utilization efficiency. The AdaBoost component focuses on improving model accuracy by sequentially adjusting the weights of misclassified instances during training. PCA is employed for dimensionality reduction, simplifying the dataset while preserving essential features that contribute to trust evaluation. The implementation involves training the AdaPCA model on the Google Cluster Usage Traces Dataset, allowing the algorithm to learn from historical data. This process facilitates the identification of patterns in user behavior and resource consumption. Once trained, the model is capable of making real-time predictions regarding trust scores and resource allocation decisions, ultimately leading to more efficient cloud resource management.

### Practical implications

The practical implications of implementing a trust-based access control framework using the AdaPCA strategy are significant for cloud computing environments. Organizations can expect improved security through the continuous assessment of user trustworthiness, which mitigates the risks associated with unauthorized access. Additionally, the efficient allocation of cloud resources leads to cost savings and enhanced performance. By optimizing resource utilization, organizations can reduce waste and improve the overall operational efficiency of their cloud infrastructure. Furthermore, the adaptive nature of the AdaPCA algorithm allows for scalability, making it suitable for various cloud environments and workloads. This flexibility enables organizations to respond to changing demands effectively while maintaining robust security measures.

### Performance under varying workload conditions

The performance of the proposed AdaPCA algorithm was evaluated under three distinct workload conditions—Low Load, Moderate Load, and High Load with Bursts—to assess its stability and efficiency in real-world cloud environments. The results were measured based on key performance indicators including trust score prediction accuracy, resource utilization, allocation time, execution time, classification metrics, and confusion matrix outcomes.

#### Low load conditions

In the low-load scenario, where job inflow was steady and contention for resources was minimal, AdaPCA consistently outperformed all benchmark algorithms. As shown in Table 3, the trust score prediction accuracy for AdaPCA during initial iterations ranged from 93.2 % to 95.4 %, while competing models such as GBM, KNN, and DT achieved lower accuracy values (ranging from 89.5 % to 91.3 %). Resource utilization for AdaPCA reached 87.2 % by the third iteration (Table 4), compared to 82.3 % for GBM and 77.1 % for KNN. Resource allocation time for AdaPCA was measured at 210 ms in iteration 3, significantly lower than KNN (290 ms) and LSTM (295 ms) (Table 5). Execution time also remained optimal, registering 1.9 s compared to 2.9 s for GBM and 3.4 s for LSTM (Table 6). These results demonstrate that AdaPCA delivers consistent and efficient performance under low-stress conditions.

#### Moderate load conditions

During the moderate workload scenario, where the job submission rate increased and task complexity varied, AdaPCA continued to maintain high accuracy. In iteration 6, AdaPCA achieved a trust prediction accuracy of 98.1 % (Table 3), while GBM and SPCA recorded 93.2 % and 93.5 % respectively. Resource utilization by AdaPCA improved to 91.0 %, whereas GBM and KNN remained below 85 % (Table 4). Furthermore, resource allocation time for AdaPCA decreased to 180 ms, outperforming all comparison models (Table 5). Execution time was optimized to 1.6 s, which was considerably lower than 2.4 s for GBM and 3.0 s for LSTM (Table 6). High precision (99.2 %), recall (99.4 %), F1-score (99.3 %), and ROC-AUC (99.6 %) further verified the reliability of AdaPCA under increased system pressure (Table 7).

#### High load with bursts

In high-load conditions with abrupt surges in job requests, AdaPCA exhibited remarkable resilience and adaptability. At iteration 10, the algorithm reached a trust score prediction accuracy of 99.8 %, the highest among all tested models (Table 3). In contrast, KNN and DT recorded only 92.3 % and 93.8 % respectively. AdaPCA achieved the highest resource utilization efficiency of 95.0 %, significantly ahead of GBM (88.2 %) and KNN (83.7 %) (Table 4). Resource allocation time was reduced to 140 ms for AdaPCA, compared to 225 ms for KNN and 200 ms for LSTM (Table 5). Execution time dropped to 1.2 s, further emphasizing the algorithm's capability to manage high-volume tasks efficiently (Table 6). Performance metrics under this condition remained excellent, with AdaPCA achieving 99.3 % F1-score, 99.6 % ROC-AUC, and strong precision-recall balance (Table 7). The confusion matrix results (Table 8) further validated the superior classification accuracy under extreme conditions. AdaPCA registered 9885 true positives and 9872 true negatives, with only 65 false positives and 78 false negatives, whereas the next best model, LSTM, had 102 false positives and 117 false negatives.

#### Adaptive trust-based scheduling

The AdaPCA algorithm was designed to dynamically adjust to workload fluctuations by recalibrating trust thresholds and re-prioritizing tasks based on predicted trustworthiness. This adaptive strategy prevented performance degradation during workload spikes and ensured optimal use of available computing resources. The algorithm’s integration of supervised dimensionality reduction with AdaBoost allowed for more focused and accurate trust evaluation, thereby enhancing both scheduling decisions and system responsiveness.

### Real-World case scenario analysis

To validate the practical applicability of the proposed AdaPCA algorithm in real-time environments, a specific case scenario was derived from the Google Cluster Usage Traces. This dataset contains job-level execution logs collected from a large-scale production data center, offering rich insights into actual workload behavior, resource demands, and system utilization patterns.

### Scenario: adaptive trust-based scheduling for interactive web services

In this case study, a continuous stream of interactive web service requests was modeled using a subset of the Google trace dataset. These services required low-latency responses and dynamic resource provisioning. Jobs associated with user-facing services—such as front-end request handling, caching operations, and small-scale backend computations—were identified based on CPU and memory usage patterns, duration, and priority labels. The AdaPCA algorithm was applied to dynamically predict the trustworthiness of resource requests by evaluating historical behavioral patterns extracted from the trace logs. Features such as average CPU usage, memory footprint, scheduling class, frequency of task failure, and time between job submissions were used to calculate trust scores. Jobs with consistent behavior and low failure rates were assigned higher trust values, allowing them to be prioritized during resource allocation.

During the execution of this scenario, the system encountered a sudden workload spike caused by an external surge in user requests. The AdaPCA model efficiently adapted to the situation by re-ranking jobs based on updated trust scores and reallocating resources without manual intervention. Compared to traditional models like Decision Trees and KNN, AdaPCA reduced the average job response time by 18.7 % and improved resource utilization efficiency by 22.4 %, as observed in the simulation. Furthermore, the integration of trust metrics ensured that malicious or misbehaving jobs—which previously led to task failures or excessive resource consumption—were deprioritized or isolated. This enhanced the overall system reliability and prevented service degradation under high-load conditions. The results demonstrated that the AdaPCA model is not only effective under controlled simulations but also exhibits strong performance in real-world dynamic cloud environments. The ability to make real-time trust-aware scheduling decisions led to improved service quality, reduced overhead, and optimized resource consumption.

### Dimensionality reduction's effect on trust factor interpretation

Dimensionality reduction techniques, particularly Supervised PCA (SPCA), play a pivotal role in enhancing the interpretability of trust factors in the proposed AdaPCA algorithm. By reducing the feature space to only the most relevant components, SPCA effectively removes noise from the dataset, ensuring that the trust prediction models rely on the most significant and informative features. This process not only improves the accuracy of trust score predictions but also makes the interpretation of these factors more transparent.

In the trust-based systems, understanding which features influence trust decisions is crucial for system administrators and users. Dimensionality reduction aids in identifying these critical features by focusing on the principal components, which are directly correlated with trustworthiness assessments. The inclusion of SPCA led to a more efficient processing of trust scores, as it achieved a higher accuracy, as opposed to models like GBM and KNN, which demonstrated lower prediction accuracy. Moreover, the reduced feature set also facilitates quicker analysis and decision-making, making trust-based systems more efficient and easier to scale.

### Critical analysis of potential limitations

Although AdaPCA demonstrates impressive performance, several potential limitations need to be addressed. One such limitation is the dependency on the quality of the input features. Since AdaPCA relies heavily on accurate and meaningful feature extraction, any inaccuracies or biases in the dataset could directly affect the algorithm’s ability to correctly assess trust levels. For instance, in real-world applications where data can be noisy or incomplete, the algorithm's performance might degrade, as indicated by the reduced accuracy observed in some alternative models such as KNN and DT.

Another limitation is the scalability of AdaPCA in large-scale systems. As the complexity of the system grows, the time taken for training and resource allocation could increase, especially if the number of features is large. Although AdaPCA performed well under various iterations, resource allocation time increased as the dataset grew larger. Furthermore, in highly dynamic environments with fluctuating workloads, the algorithm may face challenges in maintaining consistent performance without further optimization.

Finally, overfitting remains a concern with the AdaPCA algorithm when applied to specific domains. Since the algorithm optimizes its parameters based on historical data, it could struggle with unseen or highly variable data. In such cases, additional regularization techniques or cross-validation strategies could be explored to ensure robustness.

### Computational overhead in production environments

While AdaPCA offers excellent prediction accuracy, its computational overhead could pose challenges when deployed in production environments. The algorithm requires a considerable amount of computational power, especially when iterating over large datasets and performing resource allocation tasks. The execution time for AdaPCA, although relatively fast compared to other models such as LSTM and GBM, still increases with the complexity of the task.

Moreover, AdaPCA requires substantial memory resources due to its reliance on dimensionality reduction techniques and boosting mechanisms, which may increase memory usage during processing. This is particularly important in environments where computational resources are limited or expensive. While AdaPCA outperforms other algorithms in terms of resource allocation efficiency and trust prediction accuracy, the trade-off in computational demands must be considered, especially for real-time processing or large-scale cloud environments.

In production systems, optimizations such as distributed computing or hardware acceleration (e.g., GPUs) might be necessary to mitigate the computational overhead. Additionally, hybrid models could be explored, where AdaPCA is used in conjunction with lighter models to balance performance with resource utilization.

## Conclusion

The research presented demonstrates the effectiveness of combining trust-based access control with the AdaPCA algorithm for optimizing cloud resource management. The integration of trust metrics into access control decisions has been shown to enhance security and improve resource utilization efficiency. The AdaPCA algorithm, which leverages the strengths of AdaBoost and Principal Component Analysis, provides a robust framework for accurately predicting trust scores and making informed resource allocation decisions. The experimental results confirm that the proposed strategy outperforms existing algorithms, indicating its potential as a viable solution for cloud environments.

Future research directions could focus on several areas to further enhance the proposed system. First, the exploration of additional trust metrics and factors influencing user behavior could lead to more refined trust assessments. Incorporating user feedback and adapting the trust evaluation mechanism in real-time may improve the accuracy and reliability of trust scores. Additionally, examining the impact of external factors, such as network latency and security threats, on resource allocation decisions may offer a comprehensive understanding of the challenges faced in dynamic cloud environments. While the proposed AdaPCA framework demonstrates significant improvements in trust-based access control and resource optimization within cloud environments, a comprehensive cost-benefit analysis remains an essential area for future exploration. Incorporating such an analysis would quantify the tangible benefits of deploying AdaPCA in large-scale cloud infrastructures by comparing operational costs, computational overhead, and trust violation risks against the performance gains observed. Overall, these future research directions hold the potential to contribute significantly to the advancement of cloud resource optimization and security.

## Limitations

Not applicable

## Ethics statements

The paper reflects the authors' own research and analysis in a truthful and complete manner.

## CRediT authorship contribution statement

**Bala Subramanian C:** Conceptualization, Methodology, Software, Writing – original draft. **Bharathi ST:** Conceptualization, Data curation, Investigation. **Shanmugapriya S:** Visualization, Data curation, Investigation.

## Declaration of competing interest

The authors declare that they have no known competing financial interests or personal relationships that could have appeared to influence the work reported in this paper.

## Data Availability

No data was used for the research described in the article.
